# Early childhood infections, antistreptococcal and basal ganglia antibodies in adult ADHD: a preliminary study

**DOI:** 10.1186/s12888-020-02946-w

**Published:** 2020-11-18

**Authors:** Francesco Oliva, Giulia di Girolamo, Francesca Malandrone, Noemi Iaia, Fiorella Biasi, Giuseppe Maina

**Affiliations:** 1 Department of Clinical and Biological Science, University of Turin, Orbassano (TO), Italy; 2 Department of Neurosciences “Rita Levi Montalcini”, University of Turin, Turin, Italy

**Keywords:** ADHD, Group a streptococcus, Anti-streptolysin O, Anti-deoxyribonuclease B, ABGA, Basal ganglia, Adult

## Abstract

**Background:**

To explore the relationship between adult Attention Deficit/ Hyperactivity Disorder (ADHD), antistreptococcal titers, ABGA, and recurrent infections during early childhood.

**Method:**

Childhood history of recurrent infections and a blood sample were collected in a sample of DSM-IV adult outpatients with ADHD. The anti-streptolysin O (ASO), anti-deoxyribonuclease B (anti-DNase B), and anti-basal ganglia antibodies (ABGA) titers were determined in patient plasma by enzyme-linked immunosorbent assay (ELISA). Titers positivity was evaluated following manufacturer’s specifications. Absolute titers were also collected as continuous variables.

**Results:**

Fourteen out of 22 (63.6%) have had recurrent infections in childhood (i.e., seven, 31.8%, have had tonsillitis or adenoiditis and seven, 31.8%, have had any other infections). Eighteen patients (81.9%) were positive for anti-DNase B, five (22.7%) for ASO, and 4 (18.2%) were positive for both of them. Five participants (22.7%) were ABGA positive, whereas only two (9.1%) were positive for all three antibodies.

**Conclusions:**

patients with ADHD might be more prone to infections during childhood and subclinical streptococcal infections during adulthood. Moreover, they seem to have an increased risk for basal ganglia autoimmunity in adulthood. Both infections and the ensuing acquired autoimmunity could influence the neurodevelopmental process, by contributing, at least in part, to the ADHD pathogenesis.

## Background

ADHD is one of the most prevalent neurodevelopmental disorders and it is characterized by a persistent pattern of inattention and/or hyperactivity and/or impulsivity, leading to functional impairment [1] and several lifetime consequences [2]. Despite ADHD arises in childhood, it can persist in adulthood in about half of patients as a full-criteria disorder (45–57%) [3, 4], whereas a further 37% can show impairing symptoms or a subthreshold disorder at the 10 years after follow-up [3]. Neuroimaging and neurophysiological studies have consistently documented that improvement with age in core symptoms are accompanied by functional changes in cortical (right inferior frontal and inferior parietal/precuneus) and cerebellar regions [5, 6], whereas basal ganglia anomalies (i.e., right caudate) seem to be a peculiar lifetime feature of ADHD, regardless of adult remission [6, 7]. Studies on structural anomalies and age-by-diagnosis interaction in the caudate and putamen have also supported the pivotal role of subcortical basal ganglia anomalies in the pathophysiology of ADHD [8, 9].

Although the etiopathogenesis of ADHD is still unclear, a gene by environment model has been proposed to explain the high family history of the disorder [2, 10, 11]. Probably, a variable and complex interaction between predisposing genetic and environmental factors during the early months or years of life could affect the normal path of neurodevelopment. Considering the above-mentioned structural and functional evidences, environmental factors influencing basal ganglia development should be worthy of investigation. For instance, a specific vascular vulnerability of the striatum due to its positioning in a border zone of arterial supply has already been suggested by a case-control study reporting on hypoxic and other perinatal complications [12] and by a review of some in vivo studies [13]. A more recent study has documented the negative impact of exposure to polycyclic aromatic hydrocarbons on the caudate nucleus, even below the legislated annual target levels established in the European Union [14].

Over the last 20 years, the recognition of the role of some infections (e.g., group A streptococcus - GAS) in determining acute, somewhat mixed, movement (i.e., Sydenham’s chorea) and psychiatric disorders (i.e., obsessive-compulsive disorders, tic disorder, ADHD), have increased the attention to infective agents as possible causes of childhood neurodevelopmental disorders, involving basal ganglia [15–18]. A molecular mimicry mechanism has also been proposed to explain the enduring symptoms subsequent to GAS infection remission [19, 20]. Moreover, high titers of GAS infection markers, i.e., anti-streptolysin O (ASO) and anti-deoxyribonuclease B (anti-DNase B), and anti-basal ganglia antibodies (ABGA) were found in this type of disorders [21–23].

Since Kiessling and colleagues [22] have reported a higher prevalence of distractibility and hyperactivity among patients with post-streptococcal movement disorders than in controls, a growing number of subsequent studies have specifically investigated, in different ways, the relationship between GAS infections, basal ganglia autoimmunity and ADHD with conflicting results [24–28].

ADHD appears to play an important role in post-streptococcal basal ganglia disorder at least as much as obsessive-compulsive and tic disorders even when considered alone: higher titers of GAS infection markers were found in children with ADHD than in those with obsessive-compulsive or tic disorder; ADHD and even more hyperactivity symptoms significantly predicted anti-DNase B titer; and ASO titers were correlated with basal ganglia volume in children with ADHD [26].

More recently, three case-control studies have focused on GAS infection and ABGA titers of children with ADHD and without comorbid obsessive-compulsive/tic disorders [24, 27, 28]. Only one study found higher ABGA titers among children with ADHD than controls (30% vs. 15%, [28]), whereas higher titers of ASO and anti-DNase B were reported in children with ADHD by two out of three studies [27, 28]. All those inquiring about GAS infection collecting pharyngeal swabs reported higher rates of positive culture among children with ADHD than controls [24, 27]. Regardless of the specific strengths and limitations, they all were conducted on child populations and, taken together with above-mentioned studies, they suggest a possible predisposition of patients with ADHD to be infected by GAS, involving basal ganglia development.

To the best of our knowledge, no studies have assessed GAS antibodies and ABGA titers in adult patients with ADHD in an attempt to confirm the lifespan vulnerability of this population to GAS infection, which could also, in some cases, involve basal ganglia autoimmunity.

The aim of the present preliminary study was to explore ASO, anti-DNase B, and ABGA titers in a sample of adult outpatients with ADHD.

## Methods

This preliminary study was conducted at the adult ADHD outpatient center of the San Luigi Gonzaga University Hospital (Orbassano, Turin, Italy), between July 1, 2019 and September 1, 2019, on a consecutive sample of patients newly diagnosed with adult ADHD according to DSM-IV-TR criteria (age > 18 years). According to the outpatient service routine, all patients had been screened for adult ADHD using the Adult ADHD Self Reporting Scale (ASRS-1.1) screener, and then the DSM-IV ADHD diagnosis was confirmed through the Diagnostic Interview for Adult ADHD (DIVA 2.0). The severity of ADHD symptoms was rated by Adult ADHD Investigation Rating Scale (AISRS).

All patients were asked to participate in the study signing a written informed consent and a unique identification code was assigned to each patient, in order to maintain data anonymity and patient confidentiality. The research protocol of the present observational study was approved by the Research Ethics Committee of the San Luigi Gonzaga University Hospital (Orbassano, Turin, Italy), therefore the study was conducted in accordance with the Helsinki Declaration.

During the assessment process, information about the history of both infections and psychiatric disorders were collected and patients were also assessed for other psychiatric disorders through an in-depth psychiatric interview. Recurrent infections in childhood (i.e., under 12) were defined as at least three symptomatic episodes of infection with fever (e.g., tonsillitis, adenoiditis, glomerulonephritis, or others) per year for at least 1 year.

Blood samples from patients were collected in potassium ethylenediaminetetraacetic acid (EDTA) - containing tubes (BD Vacutainer® spray-coated K2EDTA, Becton, Dickinson and Company, Franklin Lakes, NJ, USA) at the end of each recruitment. Plasma was separated by centrifugation at 24,104 x g at 4 °C for 10 min and immediately stored at − 80 °C ready for analyses.

Commercial, ready-to-use, microwell ELISA kits were used for the quantitative determination of plasma concentration of ASO (Cat. No MBS038268, MyBioSource Inc., San Diego, USA), anti-DNase B (Cat. No MBS7226468, MyBioSource Inc., San Diego, USA), and ABGA (Cat. No MBS706650, MyBioSource Inc., San Diego, USA). Sample absorbance values were detected in a 96-multiwell plate reader (Model 680 Microplate Reader, Bio-Rad, Milan, Italy) using dual-wavelength recording modes at 450 nm and 655 nm (the latter used as reference). ELISA protocols instructions, and threshold values for adults provided by the manufacturer were adopted. . Thereby, the following values defined patient positivity: ASO > 200 U/ml; anti-DNase B > 86 ng/ml; ABGA > 1.18 (as the ratio referred to the negative control optical density) but absolute titers values were also collected as continuous variables.

### Clinical assessment tools

The ASRS 1.1 [29] is a six-item self-report screener for adult ADHD, based on the DSM-IV-TR criteria. It was developed by the World Health Organization (WHO) and then validated internationally [30]. The screening is considered positive when at least four answers are above cut-off value.

The DIVA 2.0 [31] is a validated [32] semistructured interview to assess the adult patients, according to the DSM-IV criteria. It investigates DSM-IV inattentive and hyperactivity/impulsivity symptoms (criterion A) in both childhood and adulthood, using several real-life examples to support each question inquiring about 18 DSM-IV criteria. It also includes explicit questions for age at onset (i.e., the presence of symptoms before the age of 7 years, criterion B) and other primary psychiatric and/or substance use disorders (which could better explain the reported symptoms, criterion E). The last section consists in an in-depth evaluation of the main areas of functioning impacted by ADHD symptoms (criteria C and D). Furthermore, the collateral information should be reported in both content and source (i.e., the presence of parents/third parties; school reports).

The AISRS is a validated [33] 18-item scale matching the DSM-IV criteria. It is divided into two subscales of nine items each, investigating inattentive and hyperactivity/impulsivity, respectively. The items are provided with examples to minimize interrater variability. The scoring system ranges from zero (none) to three (severe). The maximum score for each subscale is 27 points, with a maximum total score of 54.

### Statistical analysis

All analyses and calculations were performed using RStudio for MAC OS (Version 1.1.383, RStudio Inc., Boston, MA).

The positivity rate for every titer was calculated. The distributions of continuous variables were tested by Shapiro-Wilk’s test. A *p*-value of 0.05 was used to designate statistical significance. Mean and standard deviation (SD) were used to report normally distributed variables, whereas median (Mdn) and interquartile range (IQR) were used for nonnormally distributed ones.

## Results

A sample of 22 adult outpatients with ADHD was recruited. The sociodemographic and clinical features of the sample are summarized in Table 1. Little more than half of the patients were male. The age was not normally distributed (Shapiro-Wilk’s test W = 0.868, *p* < .001). Most of the patients have reached a middle or high school diploma, but more than a fifth of them have got a degree. Although the majority of the sample referred from GPs, psychiatrists, or child neuropsychiatrists, less than half accessed the adult ADHD center on their own (Table 1). .

**Table 1:** Sociodemographic and clinical features of the sample (*N* = 22)

Features	Value
Male, n (%)	12 (54.5%)
Age (years), Mdn ± IQR	30 ± 12
Education, n (%)	
Middle school diploma	9 (40.9)
High school diploma	8 (36.4)
Bachelor’s degree	2 (9.1)
Master’s degree	3 (13.6)
Occupational status, n (%)	
Employed	11 (50)
Unemployed	6 (27.3)
Students	5 (22.7)
Reference to ADHD outpatient center, n (%)	
Autonomous	10 (45.5)
General Practitioner	4 (18.2)
Child neuropsychiatrist	2 (9.1)
ADHD subtype, n (%)	
Predominantly inattentive	12 (54.5)
Predominantly hyperactive/impulsive	0 (0)
Combined	10 (45.5)
Positive family history of psychiatric disorders, n (%)	8 (36.4)
Positive family history of ADHD, n (%)	6 (27.3)
Psychiatric comorbidity, n (%)	8 (36.4)
Recurrent infections during childhood, n (%)	14 (63.6%)
Tonsillitis or adenoiditis	7 (31.8)
with Surgical removal	3 (13.6)
Rheumatic fever	3 (13.6)
Otitis	1 (4.5)
Mixed upperway infection	1 (4.5)
Glomerulonephritis	1 (4.5)
Pneumonia	1 (4.5)

Little more than half of the sample had a predominantly inattentive subtype ADHD. The rate of psychiatric comorbidity was high but no patient has ever suffered from tic disorder, obsessive-compulsive disorder or Sydenham’s chorea (Table 1).

Little more than a third of the sample has had recurrent infections with fever in childhood (Table 1)).

As regards antistreptococcal antibodies, 18 patients (81.9%) had positive anti-DNase B titer, five (22.7%) had positive ASO titer, four (18.2%) had them both positive. Five (22.7%) patients were ABGA positive, whereas only two (9.1%) resulted positive for all three titers. All titers were nonnormally distributed in the examined population (ASO, Median = 114.5, IQR = 82.2, Shapiro-Wilk’s test W = .792, *p* < .001; anti-DNase B, Median = 95, IQR = 17, Shapiro-Wilk’s test W = .915, *p* = .049; ASO, Median = .54, IQR = .17, Shapiro-Wilks W test = .661, *p* < .001). Within sample subgroups comparison could not be performed due to low numerosity.

## Discussion

This is the first report on anti-GAS antibodies and ABGA titers in adult patients with ADHD. Although it is a preliminary study, the magnitude of GAS infections and basal ganglia autoimmunity in adult patients with ADHD cannot be considered negligible as a large proportion of the sample was anti-DNase B positive (index of prior GAS infections), more than a quarter of the sample still showed ASO titer positivity (index of recent GAS infection) and the same amount presents ABGA positivity (index of acquired autoimmunity). The comparison with previous studies conducted on children with ADHD is challenging because of methods heterogeneity, albeit they all excluded patients with tics, obsessive-compulsive disorder, and Sydenham’s chorea. On average, the positivity rate of ASO titers reported by previous studies (ranging from 50 to 60%, [24, 28]) doubled that detected in the present preliminary investigation, even considering the well-recognized age-related decrease of the threshold value [34]. Conversely, the positivity rate of anti-DNase B in our adult sample was higher than that reported by a prior study on children with ADHD (81.3% vs. 60%, [24]). The elevation of both ASO and anti-DNase B above the threshold was found in less than half proportion of patients than that reported by two of the prior studies (ranging from 50 to 60%, [24, 27]).

All patients were recruited and tested for titers during summer, the season having the fewest GAS outbursts [34]. On the one hand, this can partially explain the lower proportion of positive ASO titers (index of recent infection) than that registered in previous studies enrolling child patients during all seasons [24, 27, 28]. On the other hand, it provides further support to our findings because they should be considered an underestimation of the actual scenario. Retrospectively collected data on childhood history of infection suggest a distinctive immune vulnerability of ADHD as almost two-thirds of the sample showed a variety of recurrent infections, half of which were tonsillitis or adenoiditis. To our knowledge, this is the first report on the history of recurrent infection in adult ADHD. Only a previous study collected this data in children with ADHD, finding that 18% (*n* = 7) of the sample had a history of GAS infections [24]. All participants of the present preliminary study were non-remitter adult ADHD, thus adults who have had ADHD in childhood but not having current ADHD were not enrolled (i.e., remitters and subthreshold ADHD patients). Bearing in mind the ADHD remission rate from childhood to adulthood (45–57%, [3, 4]), we can suppose that a history of infections and thus a possible vulnerability to them might be a common feature of persistent adult ADHD. Future studies comparing remitters and non-remitter adults should be conducted to confirm this suggestion. Furthermore, the poor reliability of retrospective data collection could partially explain this considerable discrepancy in rate history of infections between adults and children and can not allow to discern streptococcus-related from other infections.

Even more emerged about basal ganglia autoimmunity as over a fifth of the sample had positive ABGA titers, consistently with a previous report regarding Italian children with ADHD (25%, [28]). This rate was also in line with that found in adults affected by obsessive-compulsive disorder (19.8%, [35]), which represents one of the most known neuropsychiatric manifestation of post-streptococcal autoimmune basal ganglia disorders [36]. ADHD seems to be even more prevalent than obsessive-compulsive disorder in adult populations (2.8% vs. 1.2%, [4, 37]) and, as mentioned above, the implication of structural and functional anomalies of basal ganglia in its pathophysiology has been well-recognized by neuroimaging studies [5, 6, 8, 9]. However, the relationship between ADHD and post-infective basal ganglia disorders is little investigated, especially during adulthood, when the neurodevelopment is expected to be complete, thus neither infections nor the autoimmunity process can longer affect its course.

The main aim of the present study was to provide preliminary findings on the usefulness of ASO, anti-DNase B, and ABGA titers measurement in adult patients with ADHD. In this respect, we used easily available, standard, ready-to-use ELISA kit tests for the quantitative determination of blood titers that increased the reproducibility of our research and findings.

Nevertheless, different limitations should be taken into account. First of all, the sample size of the present study was small, although it was not so far from those of some clinical groups of previous case-control studies conducted on children (*n* = 20, [28] and *n* = 22, [27]), which however included a more or less appropriate control group (i.e., healthy children or patients with neurological disorders). In this regard, a noteworthy concern for future studies could be establishing the right eligibility criteria for a control group, given the impressive high psychiatric comorbidity rate of ADHD in adulthood (i.e., non-ADHD patients matched for comorbidity, enrollment season and age?). Another limitation can be recognized in current GAS infection detection because the inclusion of a rapid strep test or a pharyngeal swab culture would have been highly recommended to reach a more informative assessment.

Further studies including appropriate controls should be conducted on wider samples to confirm our preliminary findings, providing more solid evidence to support the already suggested vulnerability of patients with ADHD to some type of infections in childhood and perhaps also in adulthood [26, 28]. Nevertheless, to evaluate the trajectories of the relationship between childhood infections and ADHD development, further studies might consider ADHD remitters (i.e., adults without current ADHD but who have had ADHD in childhood). Notably, future studies may focus on a potential genetic predisposition to infection or other environmental factors affecting the neurodevelopment of the basal ganglia.

## Conclusion

According to the present preliminary study, patients with ADHD might be prone to infections during childhood, subclinical streptococcal infections in adulthood, and they seem to have a high risk for basal ganglia autoimmunity as adults. Both infections and the ensuing acquired autoimmunity may influence the neurodevelopmental process, by contributing, at least in part, to the ADHD pathogenesis. ASO, anti-DNase B and ABGA titers seem to be easy to determine by using specific ELISA kits. It may be possible that a childhood predisposition to infection, which persists into adulthood as antistreptococcal titer positivity, might also contribute to the pathogenesis of ADHD and even to its persistence. Future controlled studies should confirm on wider samples our findings on streptococcal infections, autoimmunity, and ADHD. As for some other neuropsychiatric disorders, ADHD may be related to streptococcal infections or basal ganglia autoimmunity and this could have implications on pharmacological treatment.

## Data Availability

The data that support the findings of this study are available on request from the corresponding author. The data are not publicly available due to privacy or ethical restrictions.

## Abbreviations

ADHD: Attention Deficit/ Hyperactivity Disorder
ASO: Anti-streptolysin O
anti-DNase B: Anti-deoxyribonuclease B
ABGA: Anti-basal ganglia antibodies
EDTA: Ethylenediaminetetraacetic acid
ASRS-1.1: Adult ADHD Self Reporting Scale
DIVA: Diagnostic Interview for Adult ADHD
AISRS: Adult ADHD Investigation Rating Scale
ELISA: Enzyme-linked immunosorbent assay
